# Clinical features and management of 16q24.3 microdeletion KBG syndrome: literature review

**DOI:** 10.3389/fped.2026.1742479

**Published:** 2026-02-03

**Authors:** Miaomiao Li, Shiqi Wang, Guimei Pan, Zixia Zhang, Xi Wang, Jiaqian Hu, Mengqin Wang, Mengmeng Du, Haiyan Wei, Yongxing Chen

**Affiliations:** 1 Department of Endocrinology, Genetics and Metabolism, Children's Hospital Affiliated to Zhengzhou University, Henan Children's Hospital, Zhengzhou Children's Hospital, Zhengzhou, Henan, China; 2 Pediatric Intensive Care Unit, Qingdao Women and Children’s Hospital of Qingdao University, Qingdao, Shandong, China

**Keywords:** 16q24.3, KBG syndrome, recombinant human growth hormone, short stature, treat

## Abstract

**Background:**

KBG syndrome (KBGS) is an autosomal dominant disorder presenting with diverse clinical features. Although multiple cases of the microdeletion subtype have been reported, discussions regarding its phenotypic characteristics remain relatively limited. This study aims to summarize the clinical features and management strategies for pediatric KBGS patients caused by 16q24.3 microdeletions, thereby enhancing awareness of this rare disease.

**Method:**

We conducted a retrospective analysis of the clinical manifestations, genetic characteristics, and clinical management of four pediatric patients with microdeletion-type KBGS at our institution, and systematically reviewed relevant literature to compile clinical data on affected patients.

**Results:**

All four patients exhibited typical facial features (such as cupid's bow lip, protruding ears, and thick eyebrows), skeletal abnormalities, and ocular anomalies. Whole-exome sequencing revealed a 16q24.3 microdeletion encompassing the *ANKRD11* gene. A literature review identified 68 cases (including the present cases) of KBG syndrome caused by 16q24.3 microdeletions, with a male-to-female ratio of 38:21 (9 cases of unknown sex), including 6 Chinese patients. Non-Chinese patients typically exhibit distinctive facial features including a prominent nasal root (14/28, 50%) and prominent forehead (15/33, 45.45%), whereas Chinese patients display characteristic facial features such as a cupid's bow lip, protruding ears, and thick eyebrows. Among the East Asian population (represented by Chinese individuals), the incidence of prominent eyebrows, cupid's bow lip, and delayed bone age was higher than in other populations. Patients with microdeletions involving only *ANKRD11* exhibited a higher prevalence of the characteristic triangular facial appearance and intellectual disability. In this study, the two children received recombinant human growth hormone therapy, achieving catch-up growth with height increases of 1.66 standard deviations and 0.68 standard deviations, respectively.

**Conclusion:**

The clinical phenotype of patients with microdeletion-type KBGS mainly includes characteristic facial features, macrodontia, skeletal deformities, neurological abnormalities, and eye deformities. Cupid's bow lip, protruding ears, and thick eyebrows may be characteristic facial features of Chinese children with KBGS. Genetic testing is required for definitive diagnosis. Treatment primarily relies on multidisciplinary teams providing symptomatic supportive care, with the aim of achieving early diagnosis and treatment to improve patient outcomes.

## Introduction

KBG syndrome (KBGS) (OMIM#148050) is an autosomal dominantly inherited rare disease that was first identified and reported in 1975 and was later named KBGS after the initials of the surnames of the three original families who discovered the disease (1). The *ANKRD11* gene is located on chromosome 16q24.3. Its variants and microdeletions of the 16q24.3 region, which contains the *ANKRD11* gene have been reported to be causative factors for KBGS (2, 3). The clinical manifestations of KBGS vary and include macrodontia, intellectual disability, short stature, delayed bone age, hand manifestations, cribriform vertebral anomalies, characteristic facial dysmorphisms, and the presence of this syndrome in first-degree relatives (2).

To date, multiple cases of microdeletion-type KBGS have been reported. This paper aims to summarize the phenotypic spectrum of such patients by reviewing and analyzing previously reported microdeletion-type cases, while also conducting a retrospective analysis of four pediatric microdeletion-type KBGS cases from our institution. Relevant treatment strategies for this disorder will also be discussed. The objective is to enhance clinicians' ability to recognize and diagnose the rare KBGS disorder at an early stage, thereby reducing misdiagnosis rates.

## Methods

### Study subjects

Children diagnosed with microdeletion KBGS at Children's Hospital of Zhengzhou University between July 2021 and May 2025 were included. The inclusion criteria were: (1) clinical manifestations consistent with KBG syndrome; (2) 16q24.3 microdeletion involving the *ANKRD11* gene confirmed by whole exome sequencing (WES); (3) complete clinical data and follow-up records. The exclusion criteria were: (1) KBG syndrome caused by *ANKRD11* point mutation abnormalities; (2) combined with other genetic syndromes or severe acquired diseases.

### Data collection

Data collection was conducted through a combination of medical record review and follow-up visits, encompassing multiple aspects: demographic and baseline information (patient age, gender, age at diagnosis, and family history); prenatal and perinatal history (gestational age, birth weight, intrauterine status, mode of delivery, and perinatal complications); clinical characteristics; ancillary tests (bone age assessment, growth hormone [GH] stimulation test, insulin-like growth factor-1 [IGF-1] level measurement, thyroid function tests, cranial MRI, electroencephalogram, cardiac ultrasound, and electromyography as clinically indicated), genetic testing results, and variant interpretation based on American College of Medical Genetics and Genomics (ACMG) guidelines; treatment and follow-up information was also collected, including treatment regimens (e.g., recombinant human growth hormone [rhGH] dosage, duration of use), changes in growth parameters during treatment, and adverse reactions.

### Statistical analysis

Data processing and analysis were performed using GraphPad Prism 10.1.2 software. Count data are expressed as “number of positive cases/number of total cases”. Given the small sample size of this rare disease case series, Fisher's exact test is appropriate for comparing categorical data between groups. In all analyses, a two-tailed *P*-value < 0.05 was defined as statistically significant.

### Literature review

We searched four electronic databases—PubMed, Web of Science, China National Knowledge Infrastructure (CNKI), and Wanfang Database—to summarize the clinical manifestations of microdeletion-type KBGS. The specific search terms were “KBG syndrome” for PubMed, “KBG syndrome OR 16q24.3 microdeletion” for Web of Science, and “KBG syndrome” for both CNKI and Wanfang databases. The search period spanned from the establishment of each database to May 10, 2025, aiming to collect and analyze clinical data from reported cases. Inclusion criteria were:(1)studies reporting cases of 16q24.3 microdeletion involving the *ANKRD11* gene; (2) presence of relatively well-defined clinical phenotype data; (3) full-text availability. Exclusion criteria: (1) animal studies; (2) cases lacking phenotype data or without confirmed genetic diagnosis; (3) duplicate publications.

## Results

During this period, we identified a total of 9 patients with KBGS. After excluding the KBGS patients with ANKRD11 point mutations, there were a total of 4 patients who met the criteria.

### Clinical characteristics of the 4 cases

**Figure 1:**
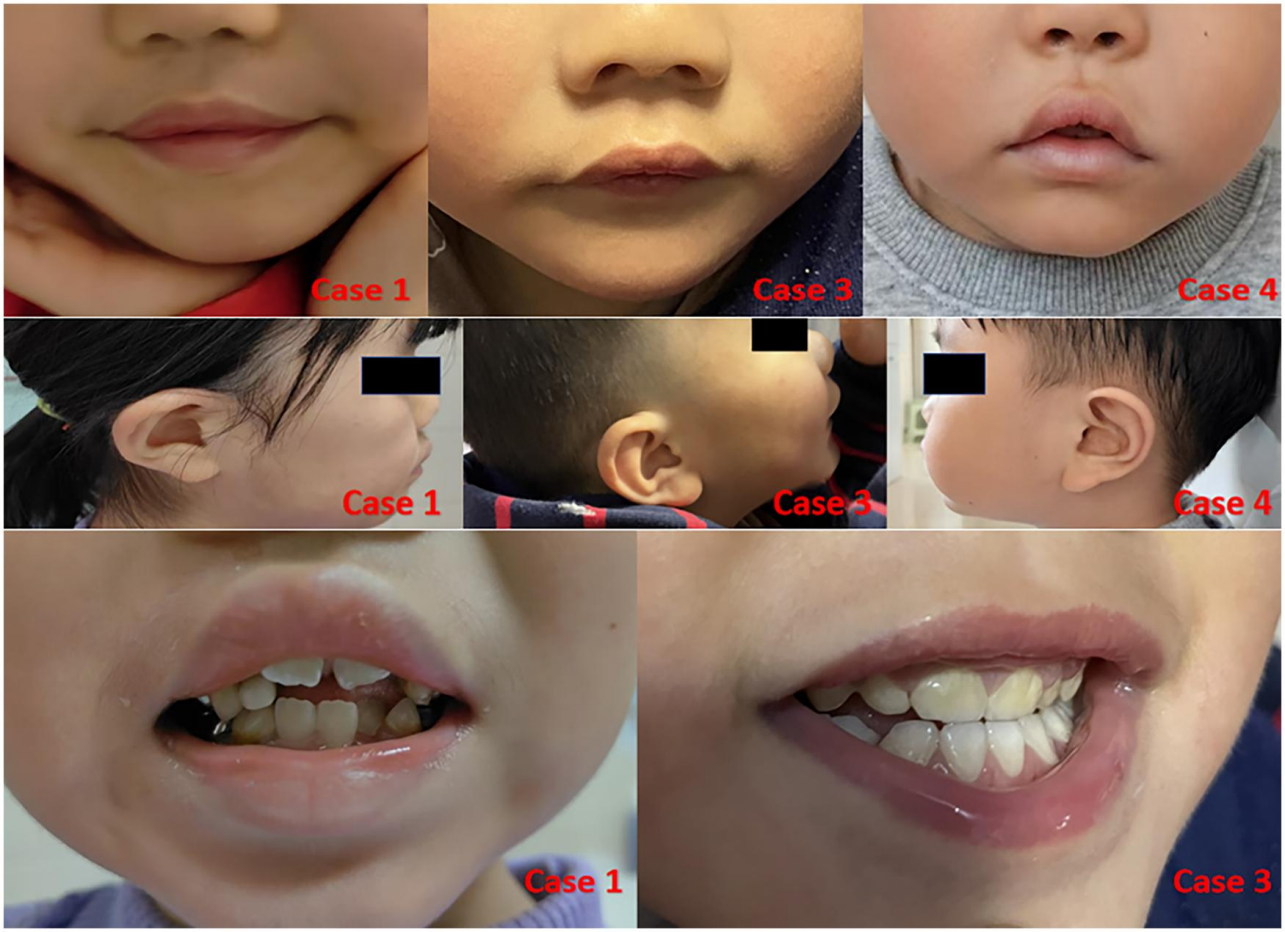
The typical features of 3 patients: case 1 was photographed at 8 years and 4 months. Case 3 was photographed at 2 years and 2 months. Case 4 was photographed at 4 years and 8 months.

The patients' detailed clinical data are shown in Table 1, and their typical phenotypic characteristics are illustrated in Figure 1. The details of the treatment with growth hormone and the follow-up situation for Case 1 and 4 are presented in Table 2.

**Table 1:** Clinical characteristics of 4 cases of microdeletion KBGS.

Data	Case 1	Case 2	Case 3	Case 4
Age, months/ Gender	100/female	19/female	26/male	56/male
Reason for initial visit	Growth retardation	Postnatal findings of short limbs	Postnatal growth retardation with ptosis	Growth retardation
Intrauterine growth restriction	**+**	**+**	**+**	**+**
Specific manifestations	Intrauterine growth restriction	Intrauterine growth restriction, thickening of the nuchal translucency, bilateral nasal bone opacity, and shortening of both humerus and femur bones	Intrauterine growth restriction	Excessive and cloudy amniotic fluid
Distinctive facial features	**+**	**+**	**+**	**+**
Thick eyebrows	**+**	**+**	**+**	**+**
Wide eye spacing	**+**	**-**	**+**	**+**
Prominent ears	**+**	**+**	**+**	**+**
Anteriorly tilted nostrils	**+**	**+**	**-**	**-**
Depressed nasal bridge	**+**	**-**	**-**	**+**
Cupid's bowed lip	**+**	**+**	**+**	**+**
Epicanthal fold	**+**	**-**	**-**	**+**
Triangular face	**-**	**+**	**+**	**-**
Ptosis of the upper eyelid	**-**	**-**	**+**	**+**
Long philtrum	**-**	**+**	**+**	**-**
Narrow palpebral fissure	**-**	**-**	**-**	**+**
Dental Abnormalities	**+**	**-**	**+**	**+**
Specific manifestations	Macrodontia, dental crowding, fused teeth	-	Macrodontia, tooth loss	Misaligned teeth, cavities
Skeletal abnormalities				
Short stature (SDS)	+(−2.17 SDS)	+(−2.10 SDS)	+(−2.03 SDS)	+(−2.33 SDS)
Delayed closure of the anterior fontanelle	**-**	**+**	**-**	**-**
Abnormal ribs	**-**	**+**	**-**	**-**
Finger abnormalities (e.g., short fingers)	**-**	**-**	**+**	**+**
Delayed bone age (Chronological age, months)	+(60)	-	-	+(28)
Neurological abnormalities				
Attention deficit hyperactivity disorder	**-**	**-**	**+**	**-**
Intellectual disability	**-**	**-**	**+**	**+** ^a^
Seizure/epileptic seizure	**-**	**-**	**+**	**-**
Delayed developmental milestones	**+**	**+**	**+**	**+**
Abnormalities on cranial MRI	**-**	Thin anterior pituitary lobe and pineal cyst	Left lateral ventricle Cyst	Pituitary gland elevated, pituitary stalk slightly deviated to the right, bilateral lateral ventricles mildly dilated, sinusitis
Other Anomalies				
Congenital heart disease	Atrial septal defect	Ventricular septal defect	-	-
Ocular abnormalities	Strabismus and amblyopia	Astigmatism	Astigmatism and hyperopia	Astigmatism
Skin and hair abnormalities	Hairy	-	Malnourished nails and transverse palm	Dry skin
Hypothyroidism	**-**	**-**	**-**	**+** ^b^
WES	16q24.2-24.3 microdeletion (chr16:88427286_89325195)	16q24.3 microdeletion(chr16:89287473_89379997)	16q24.2-24.3 microdeletion(chr16:88571621_89324308)	16q24.2-24.3 microdeletion (chr16:87981659_89416742)
Size of microdeletion	0.9 Mb	0.092 Mb	0.75 Mb	1.44 Mb
Growth hormone therapy and effects	After 2 years and 11 months of treatment, height increased from 115.8 cm (−2.04 SDS) to 141.4 cm (−0.38 SDS)	-	-	After 13 months of treatment, height increased from 99.1 cm (−2.33 SDS) to 107.5 cm (−1.65 SDS)

The facial features of the 4 patients were determined based on the standardized assessment process of the Dysmorphology Checklist (DC): Two physicians independently reviewed and cross-verified the assessments, demonstrating good agreement, ultimately confirming the typical facial phenotypic characteristics for all patients. SDS: Standard deviation score for height.

^a^ The patient underwent a Gesell developmental assessment at 4 years and 8 months of age, with results indicating a composite developmental quotient of 67.6.

^b^ This patient's thyroid function is currently normal, with a history of thyroid dysfunction.

**Table 2:** Case 1 and case 4: growth hormone therapy and follow-Up.

Data	Case 1	Case 4
Age at treatment initiation (months)	92	56
Height at the beginning of treatment（SDS）	115.8 cm (−2.04 SDS)	99.1 cm (−2.33 SDS)
Weight	19.9 kg	15.6 kg
Baseline		
Insulin-like Growth Factor 1 (IGF-1)	166 ng/mL	271.858 ng/mL
GH stimulation test peak	25.4 ng/mL	26.8 ng/mL
Initial dosage of GH administration	3.1 IU qn	3 mg qw
Follow-up		
Thyroid function	Normal	Normal
IGF-1 range	252.7-472.26 ng/mL	124.776–294.472 ng/mL
Glycated hemoglobin range	4.58–5.13%	5.39–5.66%
Glucose range	4.38–6.06 mmol/L	4.29–5.08 mmol/L
Annual growth velocity (cm/year)	8.8	7.8
Current age (months)	127	69
Current height (SDS）	141.4 cm (−0.38 SDS)	107.5 cm (−1.65 SDS)
Current Weight	36.8 kg	19kg
Current growth hormone dosage	6 IU qn	3.8 mg qw

Case 4 received long-acting GH (weekly).

The core features of the four cases of microdeletion KBGS were growth retardation and intrauterine growth restriction, accompanied by multisystem abnormalities (primarily facial features, skeletal abnormalities, neurological abnormalities, and ocular abnormalities). All four cases exhibited the characteristic facial appearance of cupid's bow lip, protruding ears, and prominent eyebrows. Three patients had dental malformations, and two presented with macrodontia. Regarding neurological abnormalities, two patients exhibited intellectual disability, with one additionally presenting attention deficit hyperactivity disorder (ADHD) and seizure episodes. Skeletal abnormalities manifested as short stature in all cases, with two patients also exhibiting short fingers, two showing delayed bone age, and one presenting with abnormal ribs. Ocular abnormalities primarily included astigmatism, strabismus, and amblyopia. Two patients also had congenital heart defects. Three patients showed cranial MRI abnormalities, and one had hypothyroidism. Two patients experienced significant improvement in height following growth hormone therapy. During follow-up, both patients maintained normal fasting blood glucose, glycated hemoglobin, and thyroid function levels (See Table 2). Microdeletion sizes varied among the four patients (0.092–1.44 Mb).

### Literature review results

Reviewing the existing literature, two cases of KBGS due to the 16q24.3 microdeletion have been reported in Hong Kong, China, together with the four patients included in this study (a total of six cases in China), three of each sex, with the age range of diagnosis from 1 month–8 years and 4 months; the size of the microdeletion ranged from 0.092 Mb to 1.44 Mb in size. A total of 98 cases of non-Chinese KBGS with microdeletion types were retrieved from the literature: 62 cases with a complete phenotype, 35:18 male:female (9 cases with unknown sex), the age range of diagnosis was 11 months–66 years, and the size of the microdeletion ranged from 378 bp to 2.3 Mb. A total of 19 non-Chinese microdeletion patients lost only the *ANKRD11* gene, and the remaining patients had mutations in other genes. Among the reported cases in China, three patients with microdeletions lacked only the entire *ANKRD11* gene. The remaining patients had deletions of other genes in addition to the *ANKRD11* gene, such as the *ZC3H18, IL17C, CYBA,* and *MVD* genes (Table 3).

**Table 3:** List of genes with deletions in the 16q24 region along with ANKRD11 in 4 patients.

Gene	Inheritance pattern	OMIM ID	Disease
*ZNF469*	AD	229200	Brittke Cornea Syndrome 1 (BCS1)
*CYBA*	AR	233690	Chronic Granulomatous Disease 4 (CGD4)
*MVD*	AD	614714	Porokeratosis 7 (POROK7)
*CTU2*	AR	618142	Microcephaly, Facial Dysmorphism, Renal Agenesis, And Ambiguous Genitalia Syndrome (MFRG)
*PIEZO1*	AR	616843	Lymphatic Malformation 6 (LMPHM6)
*CDT1*	AR	613804	Meier-Gorlin Syndrome 4 (MGORS4)
*APRT*	AR	614723	Adenine Phosphoribosyltransferase Deficiency (APRTD)
*GALNS*	AR	253000	Mucopolysaccharidosis IVA (MPS4A)
*TRAPPC2L*	AR	618331	Encephalopathy, progressive, early-onset, with episodic rhabdomyolysis
*ACSF3*	AR	614265	Combined malonic and methylmalonic aciduria(CMAMMA)
*CDH15*	AD	612580	Intellectual developmental disorder, autosomal dominant 3 (MRD3)
*ANKRD11*	AD	148050	KBG syndrome (KBGS)

**Figure 2:**
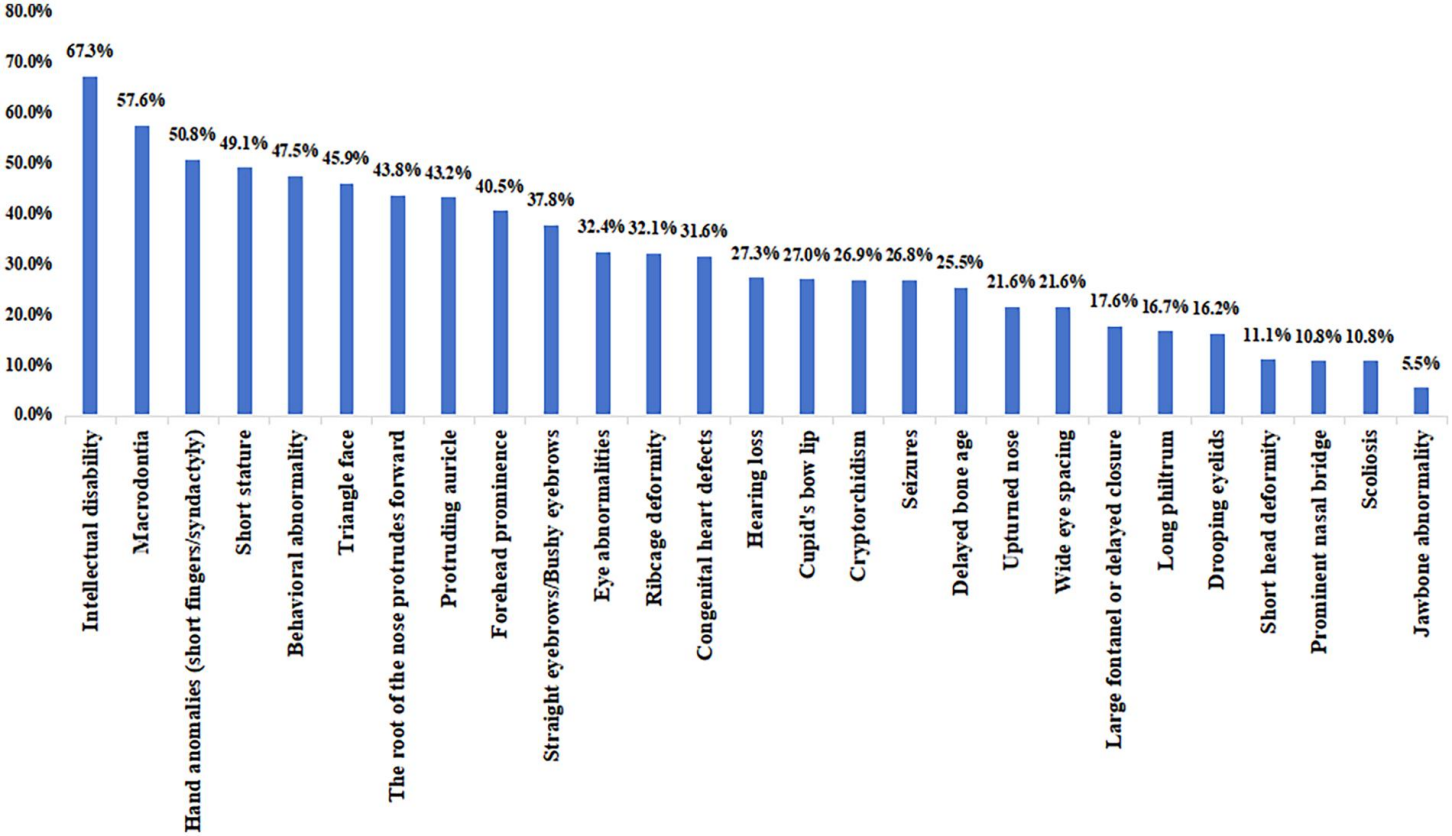
Clinical characteristics of all children with KBGS due to 16q24.3 microdeletion.

This study further analyzed the clinical phenotypes of KBGS patients with 16q24.3 microdeletions (see Tables 4, 5, and Figure 2). The core phenotypes encompass craniofacial abnormalities, skeletal deformities, neurological abnormalities, and other systemic complications, with significant variations in phenotype distribution across different populations and deletion ranges. All Chinese patients exhibited cupid's bow lip, protruding ears, and prominent eyebrows, with the incidence of these three distinctive facial features significantly higher than in non-Chinese patients (*P* < 0.05). Prominent forehead (non-Chinese: 45.45%) and prominent nasal root (non-Chinese: 50%) were more common in non-Chinese patients. Among the East Asian population (represented by Chinese individuals), the incidence of prominent eyebrows, cupid's bow lip, and delayed bone age was higher than in other populations. Short stature, delayed bone age, delayed fontanelle closure (skeletal abnormalities), and intellectual disability/developmental delay (neurological abnormalities) constitute the common phenotypes of 16q24.3 microdeletion KBGS reported in both domestic and international literature, as well as in this study. Among non-Chinese patients, epilepsy (28.85%) and cryptorchidism (29.17%) were more prevalent, but neither condition was observed in the Chinese patients included in this study. Conversely, the incidence of congenital heart defects in Chinese patients reached 50.0%, significantly higher than in non-Chinese populations. Comparing the phenotypes of patients with “*ANKRD11* gene deletion only” vs. “*ANKRD11* and surrounding region deletion”, those with *ANKRD11* deletion only showed higher rates of triangular facial features and intellectual disability (*P* < 0.05), with no significant differences in other abnormalities.

**Table 4:** Comparative analysis of clinical features in children with KBGS caused by 16q24.3 microdeletions in China and overseas, and comparison between the East Asian population and other populations.

Literature summary	1 (4)	2 (5)	3 (6)	4 (7)	5 (8)	6 (9)	7 (10)	8 (11)	9 (2)	10 (12)	11 (13)	12 (14)	13 (15)	14 (16)	15 (17)	16 (18)	17 (19)	non-Chinese KBGS total	Chinese KBGS (20)	This study	Chinese KBGS total	P*	East Asian population	other populations	P#
Total	19	4	1	2	2	1	2	1	1	1	9	3	12	1	1	1	1	62	2	4	6		9	58	
Gender (Male/Female)	10/9	4/0	1/0	1/1	1/1	1/0	1/1	1/0	0/1	1/0	/	2/1	9/3	1/0	0/1	1/0	1/0	35/18	1/1	2/2	3/3	0.6560	6/3	32/18	>0.9999
Macrodontia	9/11	0/4	0/1	0/2	2/2	1/1	2/2	1/1	1/1	1/1	3/9	3/3	4/12	1/1	1/1	1/1	/	30/53	2/2	2/4	4/6	>0.9999	7/9	27/50	0.2776
Forehead prominence	/	2/4	1/1	1/2	1/2	1/1	0/2	0/1	0/1	1/1	/	0/3	6/12	1/1	0/1	1/1	/	15/33	/	0/4	0/4	0.1186	2/7	13/30	0.6767
The root of the nose protrudes forward	/	/	0/1	1/2	0/2	1/1	0/2	0/1	0/1	0/1	/	0/3	5/12	1/1	1/1	0/1	/	9/28	/	0/4	0/4	0.3035	0/7	9/25	0.1492
Straight eyebrows/Bushy eyebrows	/	1/4	0/1	0/2	0/2	0/1	1/2	1/1	1/1	1/1	/	0/3	2/12	1/1	1/1	1/1	/	10/33	/	4/4	4/4	0.0152	7/7	7/30	0.0003
Protruding auricle	/	4/4	0/1	1/2	0/2	1/1	0/2	0/1	0/1	1/1	/	3/3	2/12	0/1	0/1	0/1	/	12/33	/	4/4	4/4	0.0276	5/7	11/30	0.2022
Cupid’s bow lip	/	0/4	0/1	2/2	1/2	0/1	0/2	0/1	1/1	1/1	/	0/3	0/12	0/1	1/1	0/1	/	6/33	/	4/4	4/4	0.0032	5/7	5/30	0.0092
Triangle face	/	3/4	1/1	2/2	2/2	1/1	0/2	0/1	0/1	0/1	/	1/3	2/12	1/1	1/1	1/1	/	15/33	/	2/4	2/4	>0.9999	3/7	14/30	0.6731
Intellectual disability	5/6	4/4	1/1	2/2	2/2	0/1	0/2	1/1	1/1	1/1	3/9	3/3	6/12	1/1	1/1	1/1	1/1	33/49	2/2	2/4	4/6	>0.9999	7/9	30/46	0.1509
Hand anomalies (short fingers/ syndactyly)	10/15	/	0/1	2/2	0/2	1/1	2/2	1/1	0/1	0/1	/	3/3	4/12	1/1	1/1	1/1	/	26/53	2/2	2/4	4/6	0.6707	6/9	24/50	0.4716
Short stature	5/9	0/4	1/1	2/2	0/2	1/1	0/2	0/1	1/1	1/1	3/9	1/3	7/12	1/1	0/1	0/1	/	23/51	1/2	4/4	5/6	0.1020	6/9	22/48	0.2973
Delayed bone age	1/2	/	0/1	0/2	0/1	/	1/2	1/1	/	1/1	/	3/3	3/12	0/1	0/1	1/1	/	11/47	/	2/4	2/4	0.2658	5/7	8/44	0.0084
Large fontanel or delayed closure	/	/	0/1	1/2	0/2	/	/	/	/	/	3/8	1/3	0/12	0/1	0/1	/	/	5/30	/	1/4	1/4	>0.9999	1/4	5/30	0.5025
Cryptorchidism	/	1/4	0/1	0/1	1/1	0/1	0/1	0/1	/	0/1	/	1/2	2/9	1/1	/	1/1	/	7/24	/	0/2	0/2	>0.9999	1/5	6/21	>0.9999
Congenital heart defects	5/19	1/3	0/1	0/2	1/2	0/1	1/2	0/1	0/1	0/1	/	1/3	5/12	0/1	0/1	1/1	/	15/51	1/2	2/4	3/6	>0.9999	4/9	14/48	0.4425
Seizures	5/18	2/4	0/1	1/2	1/2	0/1	1/2	0/1	1/1	1/1	/	0/3	2/12	0/1	0/1	1/1	1/1	16/52	/	0/4	0/4	0.3148	2/7	14/49	>0.9999

“/” indicates that the document does not mention it. “*” indicates the comparison between non-Chinese KBGS total and Chinese KBGS total. “#” indicates the population comparison between the East Asian population and other races.

**Table 5:** Comparison between KBG patients with microdeletions involving only ANKRD11 and those with microdeletions encompassing peripheral regions.

Literature summary	1	2	3	4	5	6	7	8	9	10	11	12	13	14	15	16	17	This study	Chinese KBGS	Summary	P*
Total	6/13	4/0	1/0	1/1	0/2	0/1	0/2	1/0	0/1	0/1	3/6	2/1	0/12	0/1	1/0	0/1	/	2/2	1/1	22/45	
Gender (Male)	3/7	4/0	1/0	1/0	0/1	0/1	0/1	1/0	0/0	0/1	/	1/1	0/9	0/1	0/0	0/1	/	1/1	1/1	13/25	0.7839
Macrodontia/	4/5	0/0	0/0	0/0	0/2	0/1	0/2	1/0	0/1	0/1	0/3	2/1	0/4	0/1	1/0	0/1	/	1/1	1/1	10/24	0.5447
Forehead prominence	/	2/0	1/0	0/1	0/1	0/1	0/0	0/0	0/0	0/1	/	0/0	0/6	0/1	0/0	0/1	/	0/0	/	3/12	0.3511
The root of the nose protrudes forward	/	/	0/0	0/1	0/0	0/1	0/0	0/0	0/0	0/0	/	0/0	0/5	0/1	1/0	0/0	/	0/0	/	1/8	0.2525
Straight eyebrows/Bushy eyebrows	/	1/0	0/0	0/0	0/0	0/0	0/1	1/0	0/1	0/1	/	0/0	0/2	0/1	1/0	0/1	/	2/2	/	5/9	>0.9999
Protruding auricle	/	4/0	0/0	0/1	0/0	0/1	0/0	0/0	0/0	0/1	/	2/1	0/2	0/0	0/0	0/0	/	2/2	/	8/8	0.0938
Cupid's bow lip	/	0/0	0/0	1/1	0/1	0/0	0/0	0/0	0/1	0/1	/	0/0	0/0	0/0	1/0	0/0	/	2/2	/	4/6	0.7182
Triangle face	/	3/0	1/0	1/1	0/2	0/1	0/0	0/0	0//0	0/0	/	1/0	0/2	0/1	1/0	0/1	/	2/0	/	9/8	0.0410
Intellectual disability	4/1	4/0	1/0	1/1	0/2	0/0	0/0	1/0	0/1	0/1	1/2	2/1	0/6	0/1	1/0	0/1	/	1/1	1/1	17/19	0.0092
Hand anomalies (short fingers/syndactyly)	4/6	/	0/0	1/1	0/0	0/1	0/2	1/0	0/0	0/0	/	2/1	0/4	0/1	1/0	0/1	/	1/1	1/1	11/19	0.5477
Short stature	2/3	0/0	1/0	1/1	0/0	0/1	0/0	0/0	0/1	0/1	0/3	1/0	0/7	0/0	0/0	0/1	/	2/2	0/1	7/21	0.2984
Delayed bone age	0/1	/	0/0	0/0	0/0	/	0/1	1/0	/	0/1	/	2/1	0/3	0/1	0/0	0/0	/	0/2	/	3/10	0.5208
Large fontanel or delayed closure	/	/	0/0	0/1	0/0	/	/	/	/	/	1/2	1/0	0/0	/	0/0	/	/	1/0	/	3/3	0.3858
Cryptorchidism	/	1/0	0/0	0/0	0/1	0/0	0/0	0/0	/	0/0	/	0/1	0/2	0/1	/	0/1	/	0/0	/	1/6	0.4116
Congenital heart defects	1/4	1/0	0/0	0/0	0/1	0/0	0/1	0/0	0/0	0/0	//	1/0	0/5	0/0	0/0	0/1	/	1/1	1/0	5/13	0.7709
Seizures	2/3	2/0	0/0	0/1	0/1	0/0	0/1	0/0	0/1	0/1		0/0	0/2	0/0	0/0	0/1	/	0/0	/	4/11	0.7570

“/” indicates that the document does not mention it. “*” denotes a comparison between microdeletion KBG patients involving only the *ANKRD11* gene region and those encompassing surrounding regions. All data in the tables are presented as “deletions involving only *ANKRD11* only/those including surrounding regions.”.

## Discussion

Although pathogenic variants of the *ANKRD11* gene (including inactivating mutations and deletions) have been identified as the cause of KBGS syndrome, the specific pathogenic molecular mechanisms remain incompletely understood. The protein encoded by this gene contains key functional domains: an anchor protein domain composed of five anchor protein repeat sequences, two inhibitory domains, and an activating domain (21). The *ANKRD11* gene is primarily localized within the nuclei of neurons and glial cells and plays a crucial role in neuronal migration (3). Additionally, as a key chromatin regulator, ANKRD11 may directly contribute to cognitive dysfunction in KBGS patients by modulating histone acetylation and gene expression during neural development (22). There is also evidence that neurons lacking ANKRD11 exhibit significant abnormalities in dendritic development, including reduced dendritic growth and branching, as well as altered dendritic spine morphology (23). Furthermore, functional variants in *ANKRD11* may disrupt the ordered differentiation of growth plate chondrocytes, thereby impairing longitudinal bone growth (24, 25), providing a potential explanation for the short stature phenotype commonly observed in patients.

After comprehensive analysis of the clinical manifestations of all reported patients with microdeletions (Figure 2), the clinical features of patients with KBGS due to the 16q24.3 microdeletion mainly included craniofacial anomalies such as a triangular face, prominent forehead, protruding auricle, thick eyebrow or straight eyebrow, macrodontia, skeletal anomalies such as short/parallel fingers and other hand anomalies; short stature; and costal vertebral malformation. Neurological anomalies such as intellectual disability and behavioral abnormalities of different degrees are also common. Neurological anomalies are characterized by different degrees of intellectual disability, behavioral abnormalities, etc. Other anomalies, such as congenital heart defects, eye abnormalities (astigmatism, strabismus, etc.), and hearing impairment, are also more common, and in some cases, skin and hair abnormalities and other special manifestations are also present. Previously, Prof. Luo et al. conducted a comparative study between patients with *ANKRD11* gene mutation and microdeletion KBGS, and the results revealed that children with 16q24.3 microdeletion had a greater risk of developing congenital heart defects and autism spectrum disorders and were more likely to present special facial features such as prominent forehead and triangular faces, whereas patients with the *ANKRD11* mutant phenotype have a relatively greater frequency of craniofacial abnormalities (such as large teeth deformities, long philtrum, abnormal eyebrows) and skeletal abnormalities (such as hand deformities) (26). From the perspective of genetic deletions, patients with microdeletions should exhibit more severe phenotypes than those with point mutations, as microdeletions involve larger segments of missing DNA. Our statistical analysis indicates that patients with microdeletions exclusively affecting the ANKRD11 gene are more prone to intellectual disability and characteristic facial features such as a triangular face. However, there is no significant difference in the incidence of other abnormalities like short stature. In addition, some patients with microdeletion-type KBGS also exhibit abnormal manifestations of peripheral neuropathy. In Patient 4, the child showed an abnormal gait while walking on the tiptoe during the recent follow-up, and the combination of electromyography and evoked potentials revealed that motor nerve conduction of the common peroneal nerve in the left lower limb was not evoked, that motor nerve conduction of the common peroneal nerve in the right lower limb was reduced in amplitude, that the H-wave of the H-reflexes of the tibial nerves bilaterally was also significantly reduced, and that lumbosacral spine MRI was normal, which suggests that abnormal gait may be associated with peripheral neuropathy. A previous study reported a case of 16q24.3 microdeletion KBGS in a child with abnormal stiff gait and Achilles tendon contracture as peripheral nerve abnormalities (15). However, it is not clear whether this is a rare phenotype caused by KBGS or whether the microdeletion is related to other gene deletions. Therefore, the relationships between peripheral neuropathy and KBGS and related genes need to be further investigated through the accumulation of more cases. According to the analysis of clinical data of KBGS due to the 16q24.3 microdeletion in China and abroad (Figure 2), the phenotypic spectrum of the disease shows a high degree of heterogeneity, and nonspecific or mild symptoms are often underdiagnosed or overlooked. Four (66.67%) Chinese children had dental anomalies, mainly manifested as large upper and middle incisors, malocclusion, and missing/fewer teeth, and approximately 56.60% of the non-Chinese patients had macrodontia. Macrodontia is one of the most prominent features of this syndrome and is often a clue for the diagnosis of KBG syndrome. However, in children younger than 6 years of age who have not yet developed permanent teeth, any of the signs of a specific facial phenotype, skeletal abnormalities, or neurologic abnormalities will help in the diagnosis. Moreover, there are some phenotypic differences between Chinese and non-Chinese patients (Table 4). In this study, almost all Chinese patients had cupid's bow lip, prominent auricles, and thick eyebrows, whereas nasal root prominence and forehead prominence were relatively common in non-Chinese patients; therefore, it is important to pay attention to these characteristic phenotypes in Chinese patients. There are several limitations of this study, such as the small number of cases, the varying sizes of microdeletions, and the limited ability of clinicians to capture low-frequency manifestations, which may lead to uncertainty in the results of the study; thus, more clinical cases need to be accumulated in the future.

In addition to typical clinical manifestations, children with microdeletion-type KBGS often have imaging changes and intrauterine developmental abnormalities that are of significant diagnostic value. In terms of imaging, cranial MRI abnormalities are relatively common. Previous literature reports have described 19 cases of non-Chinese patients with abnormal brain MRI findings, among which corpus callosum hypoplasia and cerebellar vermis hypoplasia were relatively common (5, 11). In this study, three patients also exhibited MRI abnormalities. These findings indicate that cranial imaging is crucial for children with suspected or confirmed microdeletion-type KBGS. Not only does it help to clarify the development of intracranial structures, but the characteristic abnormalities it reveals also provide important imaging evidence for the diagnosis of this disease. In terms of intrauterine development, Intrauterine growth restriction (IUGR) is a common prenatal phenotype. All four patients included in this study and the eight non-Chinese patients reported in the literature exhibited intrauterine growth abnormalities. The presence of IUGR during pregnancy, combined with prenatal ultrasound monitoring results and the typical clinical manifestations of the patients after birth, provides important clues for clinicians to identify and diagnose KBGS syndrome more promptly and accurately.

KBGS presents certain complexities in clinical diagnosis due to its phenotypic overlap with conditions such as DiGeorge syndrom (OMIM#188400), Sotos syndrome (OMIM#117550), Noonan syndrome (OMIM#601321) (27–29). This phenotypic similarity means that relying solely on clinical manifestations can easily lead to misdiagnosis or missed diagnosis. Therefore, definitive diagnosis of KBGS relies on molecular genetic testing, specifically confirming the presence of pathogenic variants in the *ANKRD11* gene or deletions of the 16q24.3 region containing this gene. In clinical practice, when patients exhibit characteristic combinations of symptoms (such as cupid's bow, protruding ears, thick eyebrows, and macrodontia), KBGS should be strongly suspected, and early molecular biological testing is strongly recommended. Early and accurate molecular diagnosis is key to achieving effective differential diagnosis and timely initiation of individualized interventions, which can significantly improve patient outcomes.

**Figure 3:**
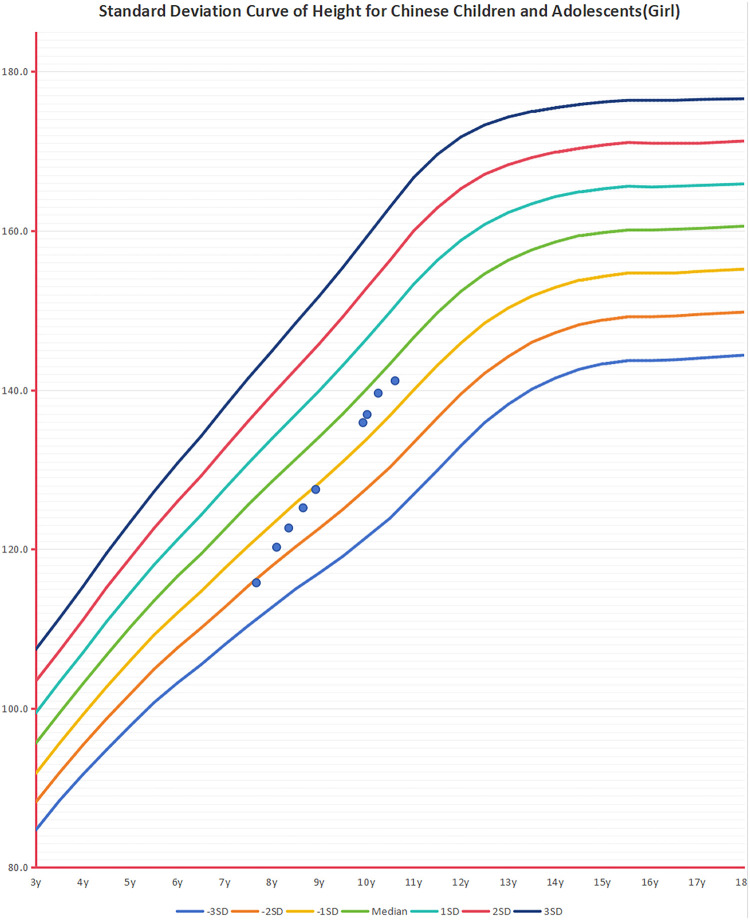
Changes in height during treatment with rhGH in case 1.

**Figure 4:**
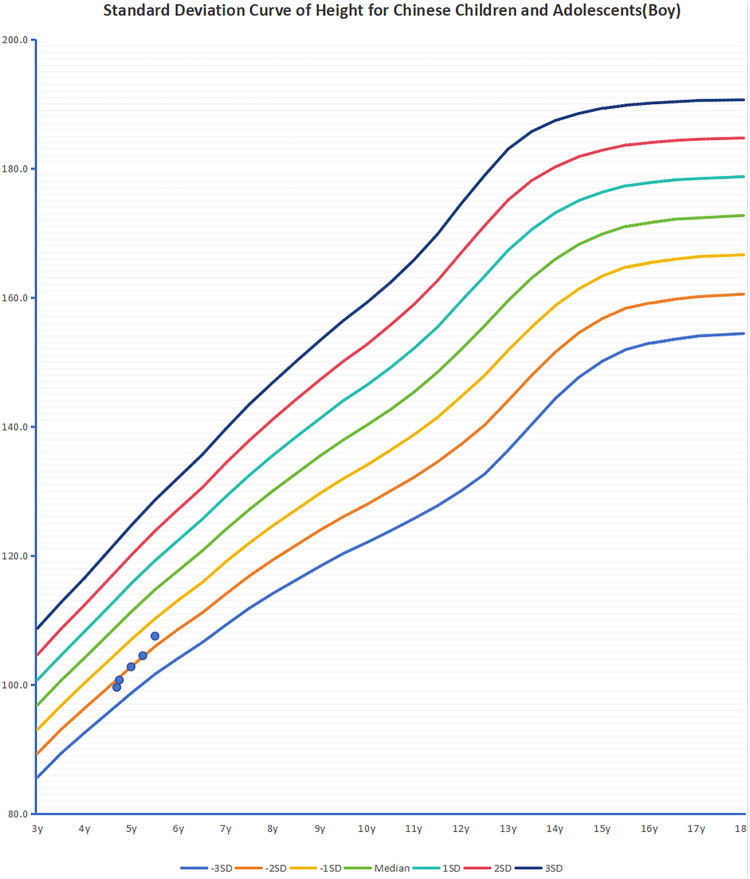
Changes in height during treatment with rhGH in case 4.

Currently, there are no specific treatment methods for KBGS. The clinical management of this condition primarily involves comprehensive supportive strategies, with the core focus on establishing and relying on the close collaboration of a multidisciplinary team (MDT). Through a comprehensive assessment of the patient, the team identifies specific challenges the individual faces in areas such as intellectual/developmental delays, behavioral issues (e.g., ADHD, autism), epilepsy, skeletal abnormalities (e.g., scoliosis), cardiac defects, feeding difficulties, and vision/hearing problems. Based on this assessment, the team develops and implements individualized, symptom-targeted supportive treatment plans. For example, patients with congenital heart defects may undergo early surgical intervention; epilepsy patients may receive early antiepileptic treatment. The objectives of these interventions are to maximize improvements in clinical symptoms, enhance self-care abilities, and improve overall quality of life. Short stature is a common symptom in KBGS patients; 83.33% of the six Chinese patients and approximately 45.10% of non-Chinese patients exhibit short stature, with spontaneous catch-up growth being limited after childhood (30, 31). In recent years, recombinant human growth hormone(rhGH) therapy has been shown to improve patient height. Previous literature reported two children with KBGS caused by ANKRD11 gene mutations, accompanied by idiopathic short stature and growth hormone deficiency, who achieved their genetic target height after rhGH therapy (30). A patient with microdeletion-type KBGS increased in height by 1.0 SDS after approximately 4 years of rhGH treatment (14). In this study, patients 1 and 4 received rhGH treatment for 2 years and 11 months or 13 months, respectively, and their heights increased by 1.66 SDS and 0.68 SDS, respectively (Figures 3,4). No abnormalities in blood glucose levels or other adverse reactions were observed during treatment or follow-up. However, given that the *ANKRD11* gene interacts with the p53 protein and may have potential tumor-suppressing effects, special attention should be paid to the safety of growth hormone therapy, particularly regarding carcinogenic risks (32). Therefore, patients receiving growth hormone therapy should undergo long-term follow-up to systematically assess treatment efficacy and social value, ensuring that treatment benefits outweigh potential risks.

## Conclusion

Children with KBGS caused by 16q24.3 microdeletion primarily exhibit distinctive facial features, macrodontia, skeletal deformities, neurological abnormalities, and ocular abnormalities. Distinctive facial features, such as a cupid's bow lip, prominent ears, and thick eyebrows, may be unique clinical manifestations in Chinese children with KBGS. Diagnosis of the condition relies on genetic testing. Treatment primarily depends on multidisciplinary diagnostic and therapeutic team collaboration, with symptomatic supportive therapy aimed at improving patient symptoms.

## Data Availability

The original contributions presented in the study are included in the article/Supplementary Material, further inquiries can be directed to the corresponding author.

## References

[1] Herrmann J Pallister PD Tiddy W Opitz JM. The KBG syndrome-a syndrome of short stature,characteristic facies,mental retardation,macrodontia and skeletal anomalies. Birth Defects Orig Artic Ser. (1975) 11(5):7–18. 1218237

[2] Ockeloen CW Willemsen MH De Munnik S Van Bon BW De Leeuw N Verrips A Further delineation of the KBG syndrome phenotype caused by ANKRD11 aberrations. Eur J Hum Genet. (2015) 23:1176–85. 10.1038/ejhg.2014.253 25424714 PMC4538199

[3] Sirmaci A Spiliopoulos M Brancati F Powell E Duman D Abrams A Mutations in ANKRD11 cause KBG syndrome, characterized by intellectual disability, skeletal malformations, and Macrodontia. Am J Hum Genet. (2011) 89:289–94. 10.1016/j.ajhg.2011.06.007 21782149 PMC3155157

[4] Goldenberg A Riccardi F Tessier A Pfundt R Busa T Cacciagli P Clinical and molecular findings in 39 patients with KBG syndrome caused by deletion or mutation of *ANKRD11*. American J of Med Genetics Pt A. (2016) 170:2847–59. 10.1002/ajmg.a.37878 27605097

[5] Willemsen MH Fernandez BA Bacino CA Gerkes E De Brouwer AP Pfundt R Identification of ANKRD11 and ZNF778 as candidate genes for autism and variable cognitive impairment in the novel 16q24.3 microdeletion syndrome. Eur J Hum Genet. (2010) 18:429–35. 10.1038/ejhg.2009.192 19920853 PMC2987261

[6] Youngs EL Hellings JA Butler MG. ANKRD11 Gene deletion in a 17-year-old male. Clin Dysmorphol. (2011) 20:170–1. 10.1097/MCD.0b013e328346f6ae 21527850 PMC5283804

[7] Isrie M Hendriks Y Gielissen N Sistermans EA Willemsen MH Peeters H Haploinsufficiency of ANKRD11 causes mild cognitive impairment, short stature and minor dysmorphisms. Eur J Hum Genet. (2012) 20:131–3. 10.1038/ejhg.2011.105 21654729 PMC3260937

[8] Sacharow S Li D Fan YS Tekin M. Familial 16q24.3 microdeletion involving *ANKRD11* causes a KBG-like syndrome. Am J Med Genet Pt A. (2012) 158A:547–52. 10.1002/ajmg.a.34436 22307766

[9] Spengler S Oehl-Jaschkowitz B Begemann M Hennes P Zerres K Eggermann T. Haploinsufficiency of *ANKRD11* (16q24.3) is not obligatorily associated with cognitive impairment but shows a clinical overlap with Silver-Russell syndrome. Mol Syndromol. (2013) 4:246–9. 10.1159/000351765 23885231 PMC3711485

[10] Khalifa M Stein J Grau L Nelson V Meck J Aradhya S Partial deletion of *ANKRD11* results in the KBG phenotype distinct from the 16q24.3 microdeletion syndrome. American J of Med Genetics Pt A. (2013) 161:835–40. 10.1002/ajmg.a.35739 23494856

[11] Miyatake S Murakami A Okamoto N Sakamoto M Miyake N Saitsu H A *De Novo* deletion at 16q24.3 involving *ANKRD 11* in a Japanese patient with KBG syndrome. Am J Med Genet Pt A. (2013) 161:1073–7. 10.1002/ajmg.a.35661 23463723

[12] Lim J-H Seo E-J Kim Y-M Cho H-J Lee J-O Cheon CK A *de novo* microdeletion of *ANKRD11* gene in a Korean patient with KBG syndrome. Ann Lab Med. (2014) 34:390–4. 10.3343/alm.2014.34.5.390 25187894 PMC4151010

[13] Kutkowska-Kaźmierczak A Boczar M Kalka E Castañeda J Klapecki J Pietrzyk A Wide fontanels, delayed speech development and hoarse voice as useful signs in the diagnosis of KBG syndrome: a clinical description of 23 cases with pathogenic variants involving the ANKRD11 gene or submicroscopic chromosomal rearrangements of 16q24.3. Genes (Basel). (2021) 12:1257. 10.3390/genes12081257 34440431 PMC8394041

[14] Scarano E Tassone M Graziano C Gibertoni D Tamburrino F Perri A Novel mutations and unreported clinical features in KBG syndrome. Mol Syndromol. (2019) 10:130–8. 10.1159/000496172 31191201 PMC6528090

[15] Novara F Rinaldi B Sisodiya SM Coppola A Giglio S Stanzial F Haploinsufficiency for ANKRD11-flanking genes makes the difference between KBG and 16q24.3 microdeletion syndromes: 12 new cases. Eur J Hum Genet. (2017) 25:694–701. 10.1038/ejhg.2017.49 28422132 PMC5533198

[16] Behnert A Auber B Steinemann D Frühwald MC Huisinga C Hussein K KBG Syndrome patient due to 16q24.3 microdeletion presenting with a paratesticular rhabdoid tumor: coincidence or cancer predisposition? Am J Med Genet Pt A. (2018) 176:1449–54. 10.1002/ajmg.a.38724 29696793

[17] Borja N Zafeer MF Rodriguez JA Morel Swols D Thorson W Bademci G Deletion of first noncoding exon in *ANKRD11* leads to KBG syndrome. Am J Med Genet Pt A. (2023) 191:1044–9. 10.1002/ajmg.a.63119 36628575

[18] Choi Y Choi J Do H Hwang S Seo GH Choi IH KBG Syndrome: clinical features and molecular findings in seven unrelated Korean families with a review of the literature. Molec Gen Gen Med. (2023) 11:e2127. 10.1002/mgg3.2127 PMC10094073 36564961

[19] Auconi M Serino D Digilio MC Gnazzo M Conti M Vigevano F Epilepsy in KBG syndrome. Develop Med Child Neuro. (2023) 65:712–20. 10.1111/dmcn.15428 36196002

[20] Ho S Luk H Lo IFM. KBG Syndrome in a Chinese population: a case series. Am J of Med Genet Pt A. (2022) 188:1693–9. 10.1002/ajmg.a.62688 35174959

[21] Zhang A Li C-W Chen JD. Characterization of transcriptional regulatory domains of ankyrin repeat cofactor-1. Biochem Biophys Res Commun. (2007) 358:1034–40. 10.1016/j.bbrc.2007.05.017 17521611 PMC1950474

[22] Gallagher D Voronova A Zander MA Cancino GI Bramall A Krause MP Ankrd11 is a chromatin regulator involved in autism that is essential for neural development. Dev Cell. (2015) 32:31–42. 10.1016/j.devcel.2014.11.031 25556659

[23] Ka M Kim W-Y. ANKRD11 Associated with intellectual disability and autism regulates dendrite differentiation via the BDNF/TrkB signaling pathway. Neurobiol Dis. (2017) 111:138. 10.1016/j.nbd.2017.12.008 29274743 PMC5803300

[24] He D Zhang M Li Y Liu F Ban B. Insights into the ANKRD11 variants and short-stature phenotype through literature review and ClinVar database search. Orphanet J Rare Dis. (2024) 19:292. 10.1186/s13023-024-03301-y 39135054 PMC11318275

[25] Barbaric I Perry MJ Dear TN Rodrigues Da Costa A Salopek D Marusic A An ENU-induced mutation in the *Ankrd11* gene results in an osteopenia-like phenotype in the mouse mutant yoda. Physiol Genomics. (2008) 32:311–21. 10.1152/physiolgenomics.00116.2007 17986521

[26] Li Q Sun C Yang L Lu W Luo F. Comprehensive analysis of clinical spectrum and genotype associations in Chinese and literature reported KBG syndrome. Transl Pediatr. (2021) 10:834–42. 10.21037/tp-20-385 34012832 PMC8107870

[27] Baldo F Fachin A Da Re B Rubinato E Bobbo M Barbi E. New insights on noonan syndrome’s clinical phenotype: a single center retrospective study. BMC Pediatr. (2022) 22:734. 10.1186/s12887-022-03804-2 36566191 PMC9789552

[28] Altshuler E Saidi A Budd J. Digeorge syndrome: consider the diagnosis. BMJ Case Reports CP. (2022) 15:e245164. 10.1136/bcr-2021-245164 PMC8811567 35110278

[29] Neeman B Sudhakar S Biswas A Rosenblum J Sidpra J D’Arco F Sotos syndrome: deep neuroimaging phenotyping reveals a high prevalence of malformations of cortical development. AJNR Am J Neuroradiol. (2024) 45(10):1570–7. 10.3174/ajnr.A8364 39147584 PMC11448971

[30] Reynaert N Ockeloen CW Sävendahl L Beckers D Devriendt K Kleefstra T Short stature in KBG syndrome: first responses to growth hormone treatment. Horm Res Paediatr. (2015) 83:361–4. 10.1159/000380908 25833229

[31] Wang S Wei H Fu D Liu X Shen L Wu S Clinical and genetic characteristics of Keishi-Bukuryo-Gan syndrome: an analysis of 5 cases. J Zhejiang University (Medical Sciences). (2021) 50:494. 10.3724/zdxbyxb-2021-0268 PMC8714474 34704418

[32] Noll JE Jeffery J Al-Ejeh F Kumar R Khanna KK Callen DF Mutant p53 drives multinucleation and invasion through a process that is suppressed by ANKRD11. Oncogene. (2012) 31:2836–48. 10.1038/onc.2011.456 21986947

